# Erythropoietic protoporphyria linked to intricate double heterozygous mutations in the*FECH* gene: a case report and literature review

**DOI:** 10.1186/s13023-025-03860-8

**Published:** 2025-07-02

**Authors:** Hongli Xiong, Song He, Zhaoxia Yang, Ruizao Zheng, Huihong Yu

**Affiliations:** Department of Gastroenterology, the Second Affiliated Hospital of Chongqing Medical University, No. 76, Linjiang Road, Yuzhong District, Chongqing, 400010 China

**Keywords:** Erythropoietic protoporphyria, Ferrochelatase, Gene mutations, Liver complications

## Abstract

**Background:**

Erythropoietic protoporphyria is an inherited disorder characterized by mutations in the *FECH* gene, which encodes the enzyme ferrous chelatase. These mutations disrupt normal heme synthesis, leading to the accumulation of protoporphyrin in erythrocytes and other tissues. Clinical manifestations include cutaneous photosensitivity, characterized by burning and itching of the skin, and, less commonly, liver failure.

**Case description:**

A teenager presented with abdominal pain, distention, and jaundice, along with a history of suspected photosensitivity. Lab tests revealed abnormal liver enzymes. Imaging showed liver cirrhosis. Liver biopsy confirmed bile accumulation and protoporphyrin deposits. Genetic testing identified a complex heterozygous mutation in the *FECH* gene, leading to a diagnosis of EPP.

**Results:**

The patient ultimately succumbed to complications arising from decompensated cirrhosis, specifically ruptured esophagogastric fundal varices and hepatorenal syndrome. We retrospectively reviewed 98 cases of EPP reported in English literature over the past decade (2014–2024). The overall mortality rate was 4.1% (4 patients).

**Conclusion:**

Patients diagnosed with EPP may exhibit hepatic dysfunction as their primary clinical manifestation. In the evaluation of the etiology of liver dysfunction, EPP, a rare genetic disorder, should be considered. This case report describes a novel mutation in the *FECH* gene located at chr18-55226418 (c.763G > T, exon 7), thereby contributing to the expanding spectrum of known *FECH* gene mutations.

Porphyrias represent a group of metabolic disorders that arise from deficiencies in enzymatic activity within the heme biosynthetic pathway. These deficiencies result in the abnormal accumulation of porphyrins or their precursors in various tissues, including the erythrocytes, liver, and nervous system. The classification of porphyrias includes eight distinct categories, which are determined by specific enzyme deficiencies within the heme synthesis pathway. These categories include X-linked protoporphyria, δ-aminolevulinic acid dehydratase deficiency porphyria, acute intermittent porphyria, congenital erythropoietic porphyria, familial or sporadic delayed cutaneous porphyria, hereditary coproporphyria, variegate porphyria, and erythropoietic protoporphyria (EPP). EPP is characterized by impaired heme synthesis resulting from a mutation in the gene encoding ferrochelatase (*FECH*). This mutation leads to the accumulation of protoporphyrin IX (PpIX) in various tissues throughout the body, which is associated with a range of clinical symptoms. EPP is typically inherited in an autosomal dominant pattern with incomplete penetrance; however, it may also be inherited in an autosomal recessive pattern. The primary clinical manifestation of EPP is a painful photosensitivity reaction to sunlight, which can present with symptoms such as erythema, edema, and pruritus. These symptoms may persist for several hours or days, and prolonged exposure to sunlight can potentially result in second-degree burns.

The management of EPP remains challenging, with limited documented cases in the existing literature. This report details a case of EPP characterized by intricate double heterozygous mutations in the *FECH* gene. Our investigation focuses on the clinical manifestations, familial genetic characteristics, and the diagnostic and therapeutic implications gleaned from this case.

## Clinical information

**Fig. 1:**
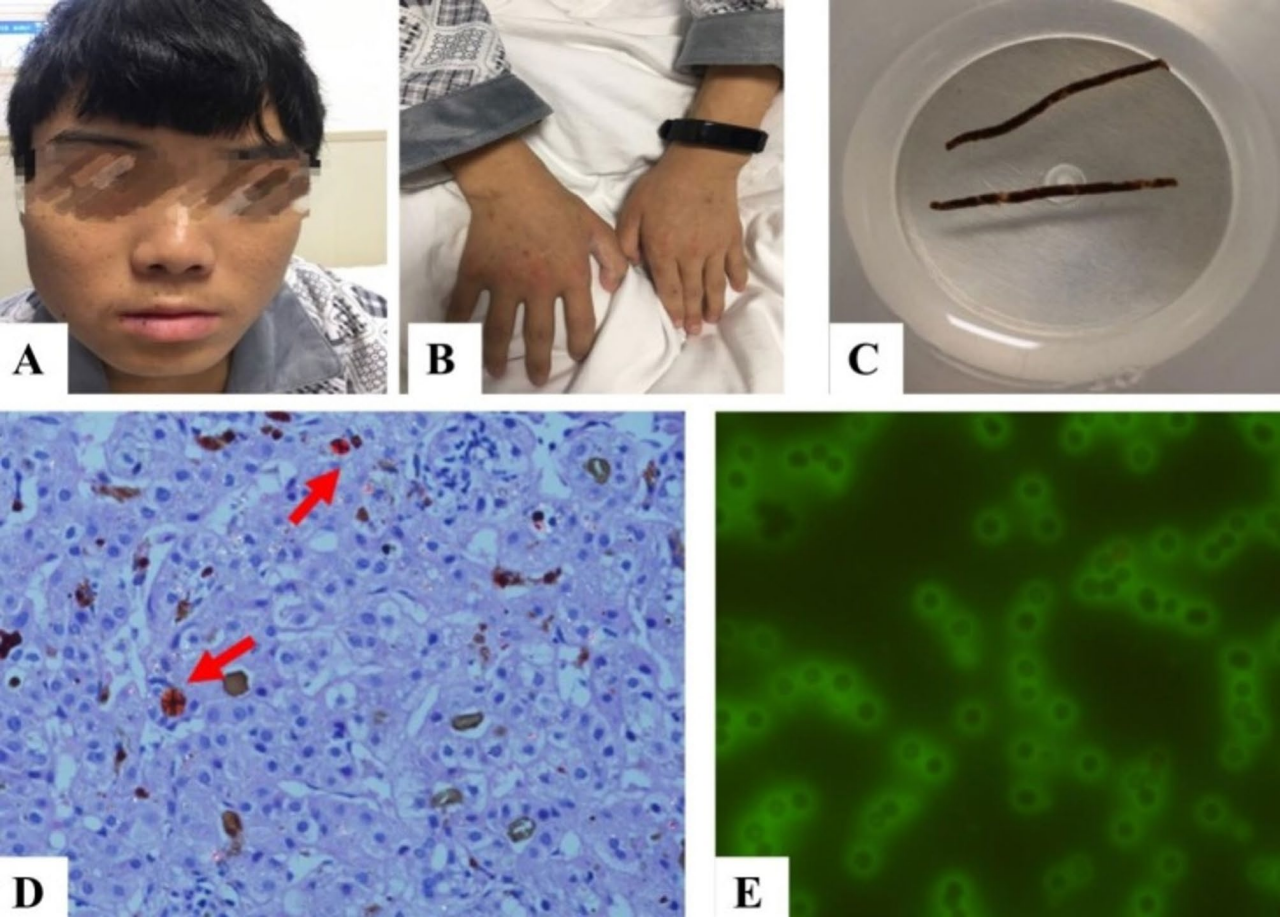
Clinical photographs of the patient showing (**A**) flaky skin with blood crusts on the lips and mouth, (**B**) slightly thickened skin on the finger joints and some lesions with blood crust formation, (**C**) liver biopsy tissue strip, (**D**) liver histopathology (**H**&**E** stain, 400×) showing pigment in Kupffer cells with birefringent crystals called “Maltese cross” configuration, (**E**) immunofluorescence of erythrocytes

**Fig. 2:**
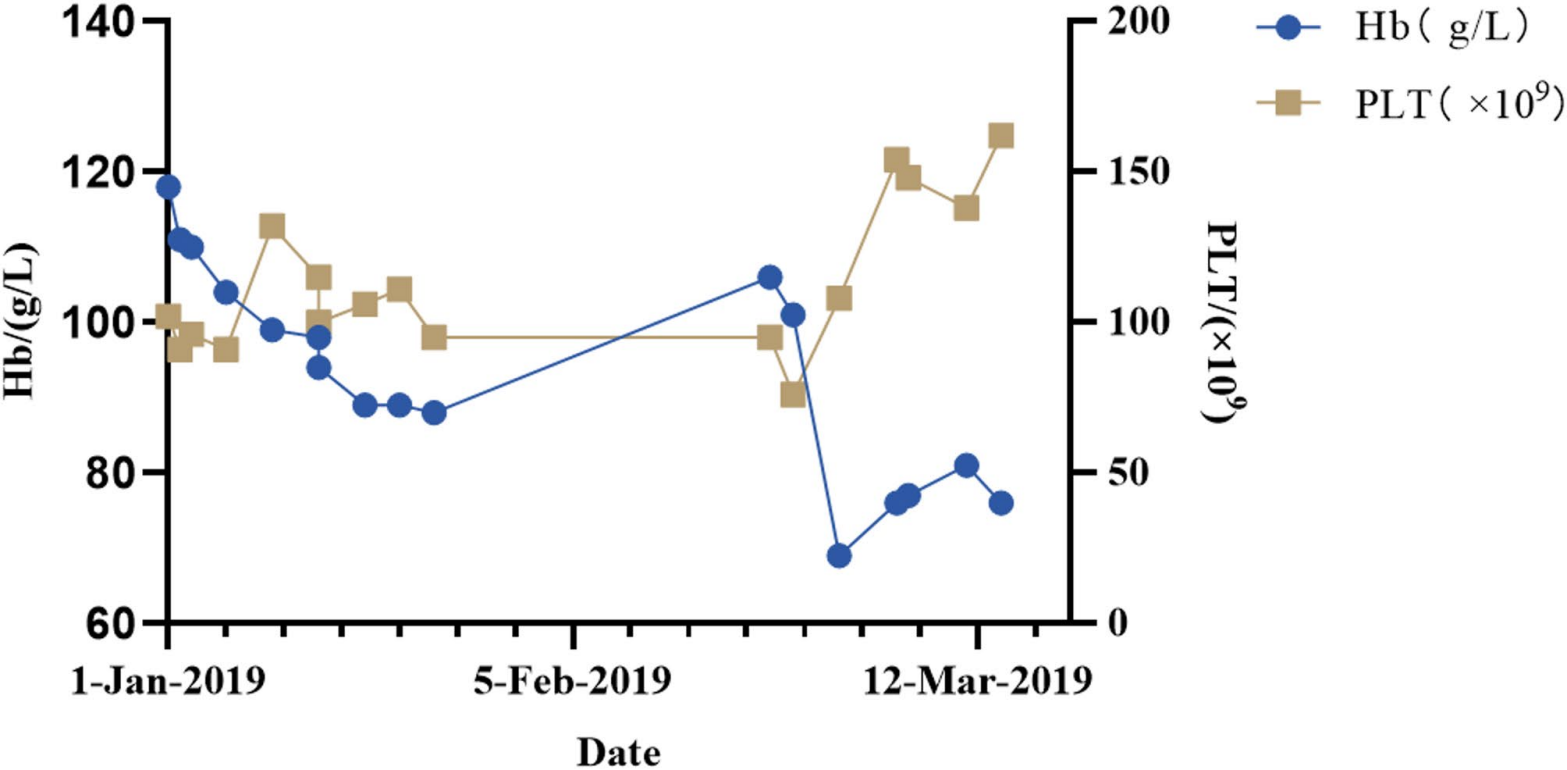
Hematological assessments of the patient. Abbreviations: Hb, haemoglobin, PLT, platelet

**Fig. 3:**
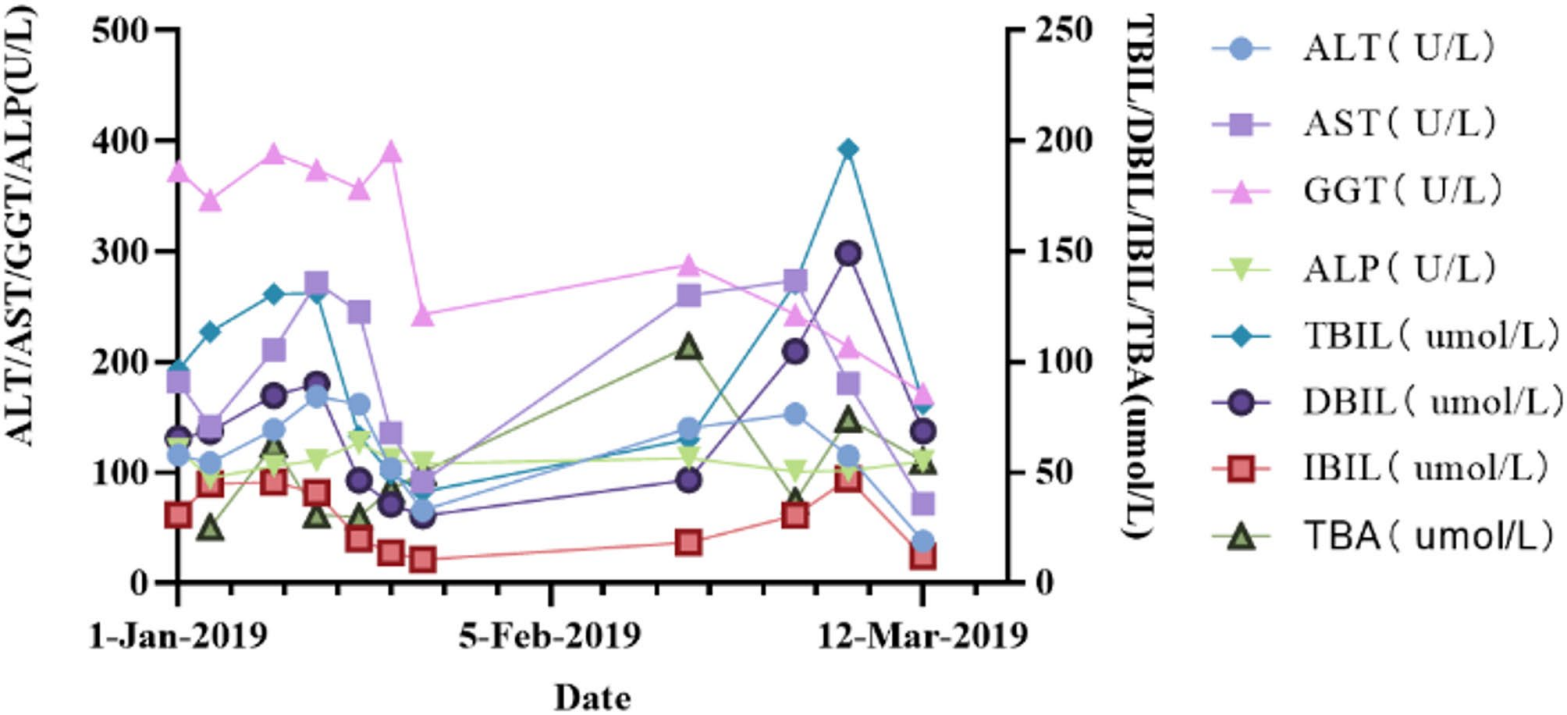
Biochemical tests of the patient. Abbreviations: ALT, alanine aminotransferase; AST, aspartate aminotransferase; GGT, gamma-glutamyl transferase; ALP, alkaline phosphatase TBIL, total bilirubin; DBIL, direct bilirubin; IBIL, indirect bilirubin; TBA, total bile acids

**Fig. 4:**
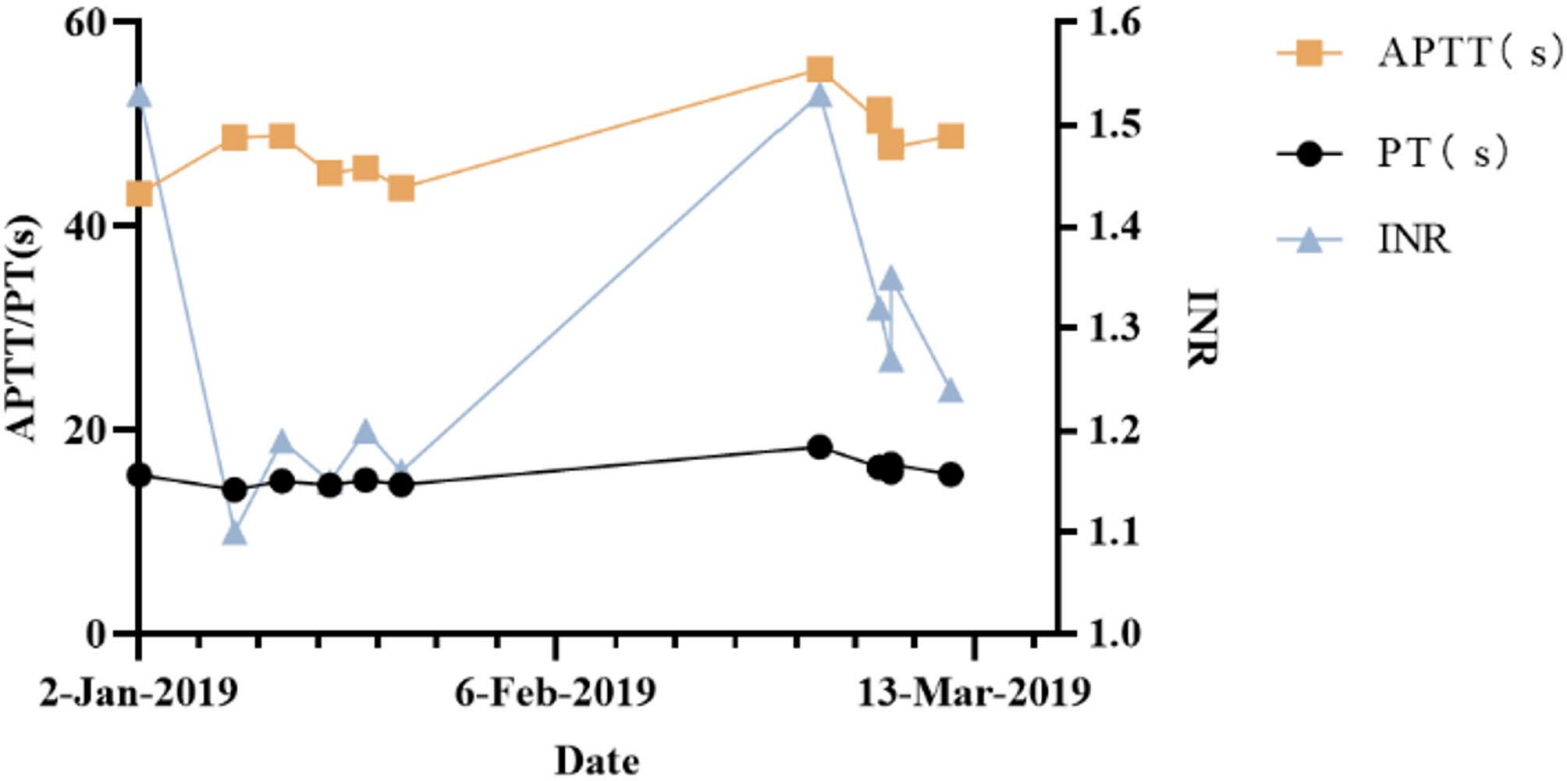
Blood coagulation of the patient. Abbreviations: APTT, activated partial thromboplastin time; PT, prothrombin time; INR, international normalized ratio

A male patient, 18 years old, presented with recurrent abdominal pain, abdominal distention, and scleral icterus. He reported experiencing suspected photosensitivity, characterized by a sensation of warmth in the skin following exposure to sunlight. A physical examination revealed generalized jaundice, desquamation of the oral mucosa and lips (as illustrated in Fig. 1 A-B), as well as the presence of spider nevi on both upper arms and the back. Additionally, the examination noted abdominal distention and periumbilical tenderness, although rebound tenderness was absent. The dynamics of blood counts, liver function parameters, and coagulation profiles are illustrated in Figs. 2–4. The patient’s autoimmune hepatic antibody profile tested positive for antinuclear antibodies (ANA) at a titer of 1:100, exhibiting a nuclear fine granular pattern, as well as for anti-smooth muscle antibodies (ASMA) at a titer of 1:320. The immunoglobulin complement assay indicated elevated IgG levels at 17.38 g/L, an increased kappa light chain level at 4.1 g/L, and a decreased complement C3 level at 2.06 g/L. An abdominal standing plain film revealed a low incomplete small bowel obstruction. Enhanced magnetic resonance imaging of the upper abdomen, including magnetic resonance cholangiopancreatography, demonstrated cirrhosis, splenomegaly, and portal hypertension. Histopathological examination of the liver biopsy revealed bridging fibrosis with incomplete septa, cholestasis within the liver lobules, and birefringent polarized light with a dark “Maltese cross” configuration, suggesting protoporphyrin deposition, as shown in Fig. 1 C-D. Special tests, including fluorescence microscopy, revealed fluorescence surrounding red blood cells, as depicted in Fig. 1 E.

*FECH* gene analysis revealed a complex heterozygous mutation located at position chr18-55226418, c.763G > T (exon 7). This nonsense mutation, inherited from the paternal lineage, leads to premature protein termination. Additionally, two other chromosome positions, chr18-5523882 and chr18-55247454, corresponding to c.315-48T > C (intron 3) and c.68–23 C > T (intron 1), respectively, were identified as heterozygous variants inherited from the maternal lineage; however, these variants did not alter at the amino acid level. Based on these findings, the patient was diagnosed with EPP, as detailed in Table 1. 

**Table 1:** Genetic testing results for the EPP patient

Variant origin	Gene	Chromosomal location hg19	Nucleotide change	Amino acid change	Hom/ Het	Variant type	dbSNP database	Population frequency ExAC	ClinVar database (Pathogenicity)
Father	*FECH*	Chr18-55226418	c.763G > T (exon7)	p.Glu255	Het	Stop gained	NA	NA	NA
Mother	*FECH*	Chr18-55238820	c.315-48T > C(intron3)	NA	Het	Intron	rs2272783	0.1073	pathogenic, EPP
Mother	*FECH*	Chr18-55247454	c.68–23 C > T(intron1)	NA	Het	Intron	rs2269219	0.24669	pathogenic, EPP

The patient was initially discharged following intensive therapy aimed at reducing peritoneal effusion, managing body temperature, alleviating abdominal pain, and ameliorating jaundice. This therapeutic regimen included the administration of anti-infectives, hepatoprotectives, anti-jaundice medications, acid suppressants, and gastric protectants. Despite the initial improvement, the patient required readmissions multiple times for ongoing management. He underwent two plasma exchange procedures on July 28 and August 19, 2019, respectively. Additionally, He received supplemental ursodeoxycholic acid, vitamin E, β-carotene, and other nutrients. Although his liver function showed improvement compared to his initial admission, as illustrated in Fig. 3, he ultimately expired due to complications arising from ruptured and bleeding oesophagogastric fundal varices and hepatorenal syndrome. These complications were secondary to the decompensation associated with liver cirrhosis.

## Literature review

A comprehensive search of Elsevier Journals, Web of Science, and PubMed utilizing the search terms “Erythropoietic Protoporphyria” AND “Case Report” from 2014 to 2024 identified a total of 74 articles, which collectively reported 98 cases, as detailed in Table 2. The cohort comprised 61 males and 37 females, with a median age of 20.5 years, indicating a predominance of young adults affected by EPP. Photosensitivity history was present in 92.9% of the patients. Elevated porphyrin levels were observed in 72 cases (73.5%). Hepatic dysfunction was noted in 52.0% of the cases. Anemia was present in 33.7% of the cases, which included 9 instances of microcytic hypochromic anemia. Additionally, abdominal pain and jaundice were reported in 23 cases (23.5%). Cirrhosis developed in 12 patients (12.2%). Liver failure occurred in 11 patients (11.2%). The overall mortality rate associated with EPP was found to be 4.1%.

**Table 2:** Overview of clinical features of reported EPP patients

Characteristics	Variable	*N*	%
	Total No. of patients	98	100.0
**Sex**	Female	37	37.8
	Male	61	62.2
**Age at diagnosis**,** years**	Median(min, max)	20.5(2,78)	
**Photosensitivity disorders**		91	92.9
**Abdominal pain**	Yes	23	23.5
	No	75	76.5
**Jaundice**	Yes	23	23.5
	No	75	76.5
**Anemia**	Yes	33	33.7
	No	65	66.3
Hypochromic anemia	Yes	9	9.2
**Liver dysfunctions**	Elevated	51	52.0
	Normal	47	48.0
**Hematoporphyrin**	Elevated	72	73.5
	Normal	26	26.5
**Liver cirrhosis**	Yes	12	12.2
	No	86	87.8
**Liver failure**	Yes	11	11.2
	No	87	88.8
**Liver biopsy**	Yes	25	25.5
	No	73	74.5
Typical presentation of liver biopsy^a^	Yes	19	76
**Genetic Testing**	Yes	69	70.4
	No	29	30.6
**Prognosis**	Dead	4	4.1

^a^Typical presentation of liver biopsy includes birefringent crystals and “Maltese cross” configuration of crystals

## Discussion

Porphyrias are inherited metabolic disorders caused by enzyme deficiencies in heme biosynthesis, resulting in porphyrin or precursor accumulation in tissues. EPP is a chronic, cutaneous form of porphyria [1]. The prevalence of EPP is estimated to be 5–15 per million, with a relatively high occurrence rate among children [2]. In a recent analysis of porphyrias within a Chinese population, the carrier rate for EPP patients was found to be 1 in 2,117, with an estimated prevalence of 7.51 per 100,000 individuals [3]. This represents the highest predicted prevalence among the various types of porphyrias. Although there is no significant difference in occurrence rates based on sex, a seasonal variation is evident, with increased incidence during the spring and summer months [4]. In this study, the male-to-female incidence ratio was found to be 2.3:1, a finding that may be influenced by the limited sample size. Diagnosing EPP can be particularly challenging due to its diverse clinical manifestations, insidious onset, and frequent involvement of multiple organ systems. The patient in this case presented with jaundice, abdominal pain, hepatosplenomegaly, and portal hypertension. While photosensitivity and skin changes were not prominent, autoimmune hepatic antibody profiling revealed positive anti-smooth muscle antibodies on two separate occasions, complicating the diagnostic process. A comprehensive history, in conjunction with genetic sequencing of porphyria-related genes, ultimately confirmed the diagnosis of EPP. EPP is primarily characterized by photosensitivity and elevated levels of plasma and erythrocyte free PpIX [5]. Pathologically, lipophilic PpIX released from erythrocytes accumulates in vascular endothelia and hepatocytes. Hepatobiliary excretion can induce cholestasis, fibrosis, and liver failure [6, 7].

The primary etiology of hepatic dysfunction in individuals with EPP is the accumulation of protoporphyrin within thehepatocytes. Consequently, the presence of protoporphyrin deposits in the liver serves as pathognomonic evidence for the diagnosis of hepatic involvement in EPP. Protoporphyrins exhibit a crystalline structure, which imparts birefringent properties when examined under a polarizing microscope, resulting in the formation of characteristic “Maltese crosses” configuration. Adjusting the angle of the polarizing filter within the microscope results in variations in both the morphology and quantity of the observed “Maltese crosses” configuration [8]. In the present case, liver histopathology with polarized microscopy confirmed protoporphyrin deposition, definitively establishing EPP-related hepatic involvement. Anemia was documented in 33.7% of patients in our literature review, with 9.2% specifically demonstrating microcytic anemia. The finding consistent in our patient, suggesting that anemia may be one of the important clinical manifestations in patients with EPP.

EPP was previously classified as an autosomal dominant disorder characterized by incomplete penetrance; however, it is now primarily recognized as following an autosomal recessive inheritance pattern. This inheritance pattern typically involves a compound heterozygous variant, which includes one mutation in the *FECH* allele (such as a deletion, missense, nonsense, or splice-site variant that results in a complete loss or significant reduction of functional enzyme activity) and a second mutation in the *FECH* gene that is associated with decreased expression. An example of such a mutation is the c.315-48T > C variant located in intron 3 [9, 10]. Additionally, the c.68–23 C > T mutation, found in intron 1, has been linked to disrupted transcriptional regulation, which results in decreased ferrous chelatase activity, altered immunohistochemical protein quantification, and reduced mRNA levels. The c.315-48T > C mutation, situated within intron 3, creates a cryptic splice site, that causing aberrant splicing of exon 4 during transcription, ultimately leading to a diminished expression of the protein product [11]. In this case, the patient’s mother presented with both the c.315-48T > C (intron 3) and c.68–23 C > T (intron 1) variants, while the father exhibited a heterozygous nonsense mutation at chr18-55226418 (c.763G > T, exon 7), which introduces a premature termination codon and activates the nonsense-mediated decay pathway. This particular mutation, which is not recorded in ClinVar or gnomAD, is classified as pathogenic according to the American College of Medical Genetics and Genomics Standards and Guidelines for the Classification of Genetic Variants (2015) [12].

Gene mutations that result in alterations of protein function can be categorized into three primary types: loss-of-function(LoF), gain-of-function(GoF), and dominant-negative mutations. While GoF mutations can significantly affect protein structure, LoF mutations lead to a complete or partial loss of protein function [13]. The enzyme *FECH*, located in the mitochondrial inner membrane, plays a crucial role in heme biosynthesis by facilitating the incorporation of ferrous ions into PpIX during the final stage of this process. Research conducted by Bonkowsky et al. and Bloomer has demonstrated that the activity of *FECH* in individuals with protoporphyria is reduced to between 10% and 25% of the normal level [14, 15]. Approximately 95% of patients with EPP exhibit a pathogenic LoF mutation in the *FECH* gene on one chromosome, in conjunction with the prevalent “low expression allele” IVS3-48 A > G on the other chromosome [16–18]. This underexpressed allele results in selective splicing, leading to a significant decrease in the expression of normal *FECH* mRNA [18]. Consequently, it is plausible that the diminished expression of the *FECH* gene in this case may be attributed to the combination of a LoF mutation and the “low expression allele”, contributing to the pathogenesis of EPP.

Existing therapies for EPP are limited in their effectiveness, necessitating strict adherence to a regimen that includes both sunlight exposure and avoidance. The diminished activity of ferrochelatase leads to the accumulation of PpIX within erythrocytes. Exposure to sunlight activates PpIX, which can lead to adverse dermatological reactions such as erythema, edema, burning, and stinging sensations. Currently, there is no effective treatment available to lower PpIX levels. However, Wulf et al. have reported that extracorporeal erythrocyte photodynamic therapy successfully reduced PpIX levels in the blood of seven EPP patients, leading to improved and “normalized” sunlight tolerance [6].

Studies have demonstrated that induced iron deficiency can enhance photosensitivity in patients with congenital erythropoietic protoporphyria, while iron supplementation has been associated with increased phototoxicity [19, 20]. Consequently, a mild iron deficiency may be beneficial for EPP patients, and iron supplementation should be considered only when symptoms of iron deficiency become severe. Additionally, β-Carotene, an antioxidant, has been shown to inhibit oxygen free radicals and reduce photosensitivity in affected patients Approved pharmacologic agonists capable of activating the melanocortin-1 receptor signaling in a targeted manner or as a bystander effect are now available for the treatment of EPP [21]. Furthermore, several pharmaceutical agents, including dersimelagon, ditopertin, ANO-V1 IVS3-48T > C, and antihistamines are currently undergoing clinical trials aimed at alleviating EPP symptoms [22–25]. These pharmacological treatments, in conjunction with therapeutic interventions such as hemoglobin infusion, plasma exchange, myelosuppression, and liver transplantation, play a significant role in the management of symptoms in patients with EPP.

Zeng et al. reported a case on EPP, wherein conventional treatments, including ursodeoxycholic acid and photoprotection measures, were found to be insufficient. In contrast, regular plasma exchange therapy resulted in a significant improvement in liver enzyme and bilirubin levels. Over a four-year follow-up period, there were no indications of disease progression or significant adverse reactions [26]. Although rare, EPP has the potential to lead to progressive liver disease. Research indicates that bone marrow transplantation may be more effective than liver transplantation in addressing the underlying enzyme deficiencies associated with EPP. Windo et al. documented a case involving an adult male patient with EPP and liver failure who underwent sequential liver transplantation followed by hematopoietic stem cell transplantation. Fifteen months post-transplantation, the patient demonstrated normal liver function [27]. Additionally, evaluations of bone marrow, peripheral blood counts, and donor chimerism confirmed the successful engraftment of the graft. The application of erythrocyte-plasma exchange in patients with EPP and liver impairment remains a contentious issue. While certain case studies indicate improvements in liver function, others argue that erythrocyte-plasma exchange, despite its efficacy in reducing protoporphyrin levels, does not necessarily enhance liver function and may not provide benefits for individuals with advanced liver disease. In the present case, the patient underwent treatment with ursodeoxycholic acid and plasma exchange throughout the course of the illness. However, the outcome was unfavorable, ultimately resulting in the patient’s death as cirrhosis progressed without liver transplantation. This case underscores the complexities and challenges inherent in the management of EPP, particularly in patients with advanced liver disease.

In conclusion, EPP is a rare condition that is often misdiagnosed or underdiagnosed. It is imperative for physicians to consider EPP in patients who present with symptoms indicative of photosensitivity and acute liver injury. Diagnosis can be confirmed through various methodologies, including clinical assessment, measurement of elevated protoporphyrin levels in erythrocytes, plasma, and feces, liver biopsy revealing the characteristic “Maltese cross” configuration, and genetic testing. This case report presents, for the first time in China, a specific mutation in the *FECH* gene (chr18-55226418, c.763G > T, exon 7). This finding broadens the spectrum of *FECH* gene mutations observed in the Chinese population, thereby enhancing our understanding of EPP in this demographic. Such insights are essential for promoting early diagnosis, implementing protective measures, and ensuring appropriate treatment interventions.

## Data Availability

The datasets generated and/or analyzed during the current study are not publicly available due to individual privacy of the patients included but are available from the corresponding author on reasonable request.

## Abbreviations

EPP: Erythropoietic protoporphyria
FECH: Ferrochelatase
PpIX: Protoporphyrin IX
LoF: Loss-of-function
GoF: Gain-of-function
